# Antigen-specific T cell Redirectors: a nanoparticle based approach for redirecting T cells

**DOI:** 10.18632/oncotarget.11785

**Published:** 2016-09-01

**Authors:** Christian Schütz, Juan Carlos Varela, Karlo Perica, Carl Haupt, Mathias Oelke, Jonathan P. Schneck

**Affiliations:** ^1^ Institute of Cell Engineering and Department of Pathology, Johns Hopkins School of Medicine, Baltimore, Maryland, USA; ^2^ Division of Hematology, Department of Medicine, Sidney Kimmel Comprehensive Cancer Center, The Johns Hopkins Hospital, Baltimore, Maryland, USA; ^3^ NexImmune Inc., Gaithersburg, Maryland, USA; ^4^ Current address: Division of Immunology, Paul-Ehrlich-Institut, Langen, Germany

**Keywords:** cancer, redirection, antigen-specific T cells, nanoparticle, MHC-Ig

## Abstract

Redirection of T cells to target and destroy tumors has become an important clinical tool and major area of research in tumor immunology. Here we present a novel, nanoparticle-based approach to selectively bind antigen-specific cytotoxic T cells (CTL) and redirect them to kill tumors, termed ATR (Antigen-specific T cell Redirectors). ATR were generated by decorating nanoparticles with both an antigen-specific T cell binding moiety, either peptide loaded MHC-Ig dimer or clonotypic anti-TCR antibody, and a model tumor cell binding moiety, anti-CD19 antibody to engage CD19^+^ tumor cells. ATR stably bind tumor cells and CTL in a dose dependent fashion and stimulate antigen-specific conjugate formation between those cells. ATR induced redirected lysis of tumor cells *in vitro*, as demonstrated by ^51^Cr-release killing. *In vivo* ATR administration led to reduced tumor growth in a SCID/beige human lymphoma treatment model. In summary, ATR represent a novel, nanoparticle based approach for redirecting antigen-specific CTL to kill tumors.

## INTRODUCTION

Antibodies and antibody-like molecules have emerged as a major, clinically important therapeutic approach for treatment of a wide variety of diseases including autoimmunity, inflammation and cancer. Part of the repertoire of antibody-like molecules under development or in use have been the generation of complexes with the potential to bind two or more targets simultaneously, such as immunotoxins, radio-immunoconjugates, bi-specific antibodies, bi-specific single-chain Fv antibodies and tandem single chain triplebodies [1–6]. Bi-specific antibody-like complexes have multiple modes of action including: (1) inhibition of two different cell surface receptors; (2) blocking of two ligands; (3) crosslinking of two receptors; (4) delivery of toxins or death inducing agents to kill tumor cells; and (5) T cell recruitment to the proximity of tumor cells to induce antibody-dependent cellular cytotoxicity, also named redirected lysis [7].

Bi-specific antibody technology designed for treatment of cancer is used clinically in the treatment of lymphoma and some types of leukemia. Currently the highly successful technology is termed ‘BiTEs’ (bi-specific T cell engagers) and Blinatumomab (MT103; Micromet/Medimmune), a BiTE specific for CD19 and CD3, is used to treat patients with non-Hodgkin's lymphoma and is also in several clinical trials for the treatment of acute lymphoblastic leukemia (ALL) [8]. These bi-specific antibodies engage T cells through use of conserved component of the TCR, such as CD3. By recruiting all T cells using anti-CD3, *in vivo* complications due to global T cell activation have been observed and necessitate careful i.v. dosing, requiring continuous infusion over weeks. Binding T cells non-specifically may also result in undesired effects that compromise efficacy. Since most T cells are not effector T cells, non-specific binding recruits irrelevant T cells to the site of interest. In addition to recruiting irrelevant T cells, it may also recruit regulatory T cells, which would inhibit effector T cell populations and further limit efficacy. In contrast selective recruiting of antigen-specific cytotoxic T cells could serve as a platform for redirecting T cells that could be effective without the associated risks attached to non-specific T cell binding.

Here we describe a novel, nanoparticle-based approach to selectively bind antigen-specific T cells and redirect them to kill tumors, termed ATR (Antigen-Specific T cell Redirectors). ATR were generated using either ^pep−^MHC-Ig dimer or anti-TCR-specific mAb to bind specific effector T cell populations. These were immobilized onto a nanoparticle along with anti-human CD19 antibody. This nanoparticle complex stably binds antigen-specific T cells and tumor cells, ensures conjugate formation between these two cells and redirects mouse and human T cells to kill human tumor cell *in vitro* and *in vivo*.

## RESULTS

### Generation of nanoparticle-based ATR

**Figure 1:**
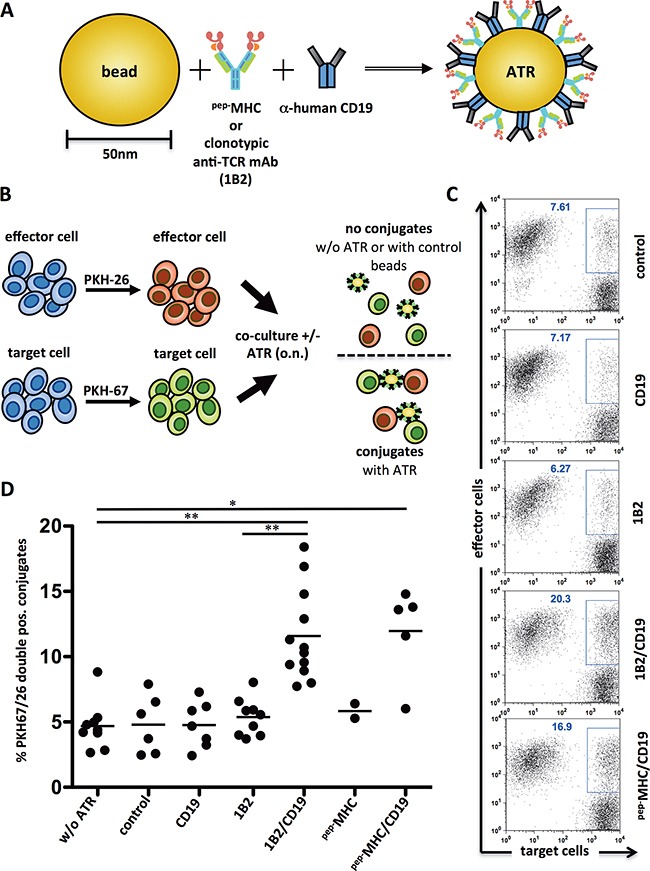
ATR induce antigen-specific conjugate formation **A.** Schematic of an ATR. Anti-mouse-IgG1 nanoparticles were coated with anti-human CD19 mAb and peptide loaded MHC-Ig molecules (^pep−^MHC) or a clonotypic anti-TCR mAb specific for the transgenic TCR of 2C T cells (1B2). For controls particles with only one signal (^pep−^MHC, 1B2 or CD19) were generated. A mouse IgG1 isotype control substituted the second signal. An additional control particle was mouse IgG1 mAb isotype control only. **B.** Schematic of an experimental set up for a conjugation assay. **C.** Flow cytometry data from one exemplary conjugate formation assay. PKH-67 stained target cells (T2) and PKH-26 stained effector cells (2C T cells) were co-cultured with indicated ATR at 4°C, o.n. at a 1:1 ratio. Control indicates particles with immobilized mouse-IgG1 isotype control only. **D.** Summary of 2-12 independent conjugation assays utilizing T2 tumor cells and 2C T cells. ^pep−^MHC indicates ATR made with SIY peptide loaded K^b^-Ig dimer. * (p<0.05) and ** (p<0.001) indicates statistical significance (Wilcoxon-Mann-Whitney-Test).

The model system used to generate nanoparticle based ATR to redirect murine CTL was the mouse 2C TCR transgenic T cells. Antigen-specific 2C T cells, which recognize the synthetic SIYRYYGL peptide (SIY) in the context of the murine MHC H-2K^b^, were recruited using either a peptide loaded MHC-Ig complex (^SIY^K^b^-Ig) or an anti-clonotypic antibody (1B2); tumor cells were recruited with an anti-human-CD19 (Figure 1A). Control nanoparticles were generated with one signal (^pep−^MHC-Ig, 1B2 or anti-CD19) only and a mouse isotype control substituted for second signal. ATR only showed binding to cells expressing relevant cognate complexes (Supplementary Information and Supplementary Figure S1).

### ATR stimulate antigen-specific effector T cell/target cell conjugate formation

ATR stimulate conjugate formation between T cells and tumor cells was studied using a flow cytometry-based assay [9]. 2C T cells and CD19^+^ T2 tumor cells were stained with a red (PKH26) and with a green fluorescent membrane dye (PKH67), respectively. Co-cultures with ATR enhanced 2C/T2 conjugate formation, represented by an increased population of PKH26/67 double positive cells (see schematic, Figure 1B). ATR (1B2/CD19 and ^pep−^MHC-Ig/CD19) increased conjugate formation from a baseline of 7.61% up to 20.3% and 16.9%, respectively (Figure 1C). Control particles (1B2, CD19) showed only conjugate formation (6.27%, 7.17%, Figure 1C) similar to background levels (7.61% control, Figure 1C). A summary of conjugate formation assays show that 2C/T2 conjugate formation is highly significant (p<0.01) in the presence of ATR when compared to controls (Figure 1D). While both antibody (1B2/CD19) and ^pep−^MHC-Ig (^SIY^K^b^-Ig) based ATR induced conjugate formation, ATR generated with an irrelevant T cells targeting complex, ^OVA^K^b^-Ig, did not show an increased PKH26/67 double positive population (data not shown).

Specificity, stability and ratio dependence of ATR binding to cell was also studied. Optimal ATR binding of up to 0.2×10^6^ 2C T cells was seen using 50 μl of ATR (5×10^9^ particles/ml) (Supplementary Information and Supplementary Figure S2A-S2D). Particle binding to cells was significantly diminished using less ATR, 1/10 or 1/100, but 50 μl of ATR could bind up to 3×10^6^ cells displaying the same efficacy. Particle-cell conjugates were stable for at least 60 min at 37°C and led to T cell activation as measured by CD107 expression and proliferation (Supplementary Information and Supplementary Figure S2E and S2F).

### ATR redirected lysis of human CD19^+^ tumor cells

**Figure 2:**
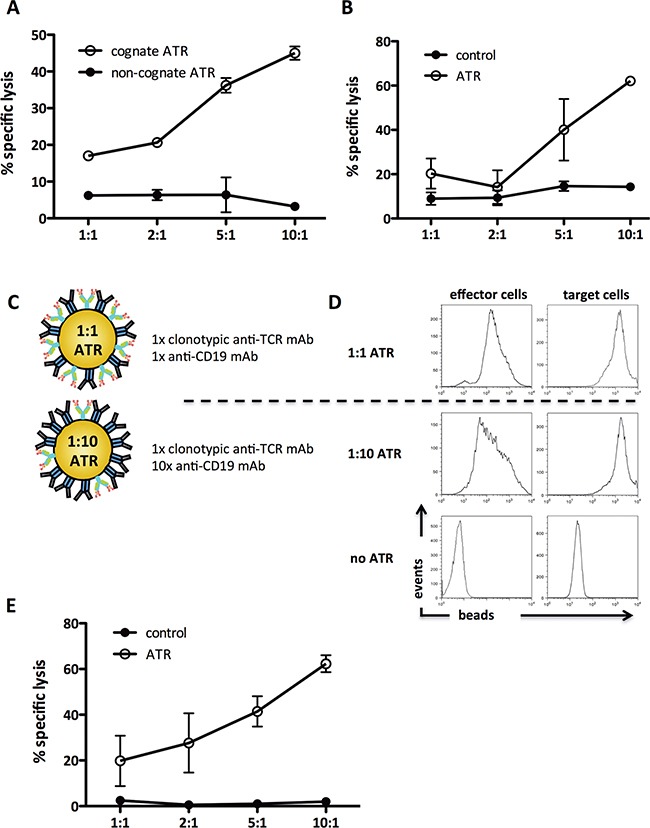
ATR redirect T cells to CD19^+^ tumor cells antigen-specific and variation of effector cell:target cell signal ratio increases ATR function Redirected killing in CD19^+^ tumor cells (Raji) by ATR was determined by ^51^Cr release assay. **A.** Antigen-specific engagement of 2C T cells with cognate ATR (^SIY−^K^b^-Ig/CD19) and non-cognate ATR (^OVA−^K^b^-Ig/CD19) was analyzed. **B.** 2C T cells mediated redirected lysis of CD19^+^ tumor cells (Raji) through ATR (1B2/CD19). To control for specific TCR engagement effects on 2C T cells in redirection cultures beads coated with 1B2 and isotype control mAb were used (control). **C.** Schematic of a 1:10 and a 1:1 ATR. **D.** Staining control of 1:1 and 1:10 ATR. Effector cells (2C T cells, upper panel) and target cells (Raji tumor cells, lower panel) were stained with ATR at 4°C for 15 minutes, washed and co-stained with anti-IgG1. **E.** 2C T cells mediated redirected lysis of CD19^+^ tumor cells (Raji) through 1:10 ATR (1B2/CD19). To control for specific TCR engagement effects on 2C T cells in redirection cultures, beads coated with 1x 1B2 and 10x isotype control mAb were used (control). All data displayed represents background subtracted (cells only) specific lysis of tumor cells, derived from triplicates of the same experiment.

Redirected killing of human tumor cells was studied by incubating ATR (^SIY^K^b^-Ig) with CD19^+^ Raji cells and 2C T cells. ATR redirected lysis of Raji cells over various Effector:Target (E:T) cell ratios tested with specific killing increasing from 10% to 42% (Figure 2A). In contrast, minimal changes were seen in background killing by non-cognate ATR (^OVA^K^b^-Ig) (Figure 2A). ATR killing efficacy was highest utilizing 5×10^9^/ml particles (Supplementary Information Figure S2G). In addition no lysis was detected in CD19 bead control samples (data not shown).

Redirected lysis was also induced by ATR which used an anti-clonotypic mAb (Figure 2B). Specific lysis was detected using 1B2/CD19 ATR over the entire range of E:T cell ratios with a maximal specific lysis of 48% (10:1). Thus both ^pep−^MHC-Ig and clonotypic anti-TCR mAb, based ATR are able to redirect lysis of human Raji B cell lymphoma cell line.

### Optimizing ATR formulation

An advantage of the nanoparticle based system is the ability to easily vary the ratio of T cell to tumor cell binding complexes. This allows one to optimize tumor cell killing while minimizing non-specific T cell activation. Based on the 1:1 stoichiometry of the bi-specific antibody technology, our initial ATR was generated using a 1:1 ratio of binding complex (Figure 2C, upper panel). Additional ATR were generated with a 1:10 ratio of T cell:tumor cell binding complexes (Figure 2C, lower panel). Therefore, the T cell binding signal was reduced and the tumor cell binding signal was kept constant. To evaluate the effective binding of ATR to their targets, 2C T cells (Figure 2D, left panel) or CD19^+^ Raji tumor cells (Figure 2D, right panel), were incubated with the different ATR formulations and stained with anti-mouse IgG1 mAb. Both ATR bound to tumor cells with comparable MFI; however ATR made with less T cell binding complexes demonstrated a reduced MFI compared to 1:1 ATR staining. A 1:100 ATR did not show any more significant binding to cells (data not shown).

Although 1:10 ATR showed less binding to 2C T cells, 1:10 ATR efficiently redirected lysis of tumor cells over the entire E:T range tested. Specific lysis increased from 18% up to 60% at an E:T of 10:1 (Figure 2E). In contrast non-specific lysis by control particles was significantly less between 0.5-2%. Thus titration of both binding complexes on ATR can be used to increase efficacy.

### ATR induce human influenza antigen-specific CTL redirected lysis of human CD19^+^ tumor cells

**Figure 3:**
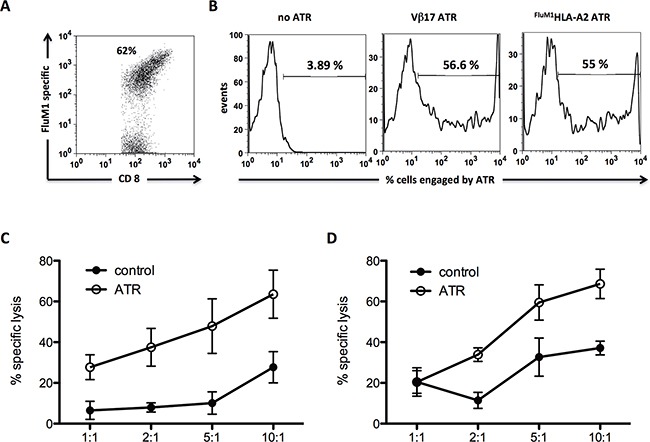
Human FluM1 specific CD8^+^ T cells can be redirected to kill CD19^+^ Raji tumor cells **A.** FluM1 tetramer stain of human FluM1 specific CTL utilized for ^51^Cr release redirection assay. **B.** Staining control of generated human ATR. Effector cells (human FluM1 specific CTL) were stained with ATR at 4°C for 15 minutes (Vβ17/CD19) or 45 minutes (^FluM1^HLA-A2/CD19), washed and secondary stained with anti-mouse-IgG1. Human FluM1 specific CTL mediated redirected lysis of CD19^+^ Raji tumor cells through Vβ17/CD19 **C.** and ^FluM1^HLA-A2/CD19 **D.** ATR. To control for specific TCR engagement effects on FluM1 specific CTL in redirection cultures, particles coated with anti-Vβ17 mAb and isotype control mAb or ^FluM1^HLA-A2 and isotype control mAb were used, respectively (control). All data displayed represents background subtracted (cells only) specific lysis of tumor cells, derived from triplicates of the same experiment.

As a model system to redirect antigen-specific human T cell lysis, we studied the ability to redirect lysis by the influenza M1-specific CTL. FluM1 specific CTL were generated from a healthy HLA-A2^+^ donor (Figure 3A) and ATR made using either FluM1 loaded HLA-A2-Ig (^FluM1^HLA-A2/CD19) or anti-Vβ17 mAb (Vβ17/CD19), which also targets the majority of this population. Flow cytometry analysis revealed that both ATR bound to CTL at comparable levels, 56.6% (Vβ17/CD19) and 55% (^FluM1^HLA-A2/CD19), respectively (Figure 3B). Binding efficiency of both ATR to CTL matched the FluM1 specificity of CTL determined by tetramer stain (Figure 3A).

ATR induced redirected lysis of CD19^+^ Raji tumor cell by FluM1 specific CTL. Specific lysis was detected between 24% - 60% (Vβ17/CD19) (Figure 3C) and from 20% - 62% (^FluM1^HLA-A2/CD19) (Figure 3D). Controls of Vβ17 and ^FluM1^HLA-A2 particles showed no redirected lysis over background levels.

### ATR treatment of B cell lymphoma in vivo

**Figure 4:**
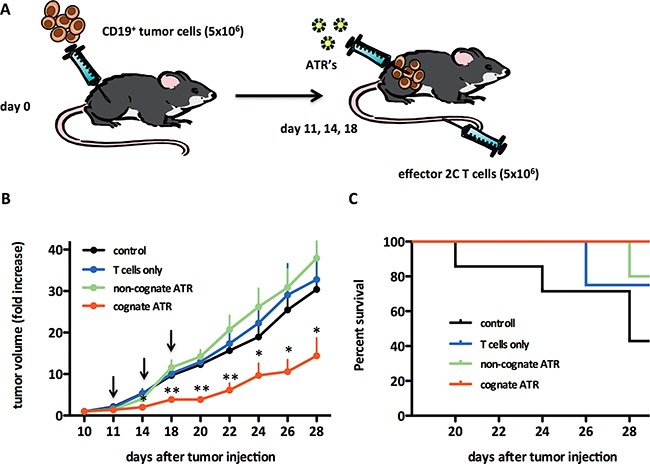
ATR reduce tumor growth *in vivo* in a Raji tumor model **A.** Schematic of the experimental *in vivo* set up. SCID/beige mice were injected on day 0 s.c. with 5×10^6^ CD19^+^ Raji tumor cells. Mice were monitored for tumor growth and tumors were measured by caliper. At day 11 mice with palpable tumor were divided into four groups; control, T cells, non-cognate (^OVA^K^b^-Ig/CD19) or cognate ATR (^SIY^K^b^-Ig/CD19). For treatment mice were adoptively transferred i.v. with 5×10^6^ activated 2C cells and 100 μl ATR were injected intra tumoral. Treatment was repeated on day 14 and 18 (black arrows). Animals from T cell groups were not treated with ATR and control animals did not receive 2C T cells and ATR. **B.** Data displayed as fold increase of tumor volume. Fold increase of tumor volume was calculated for each mouse related to tumor volume on day 10. **C.** Survival curves from groups displayed in (B). Number of animal per group: control (n=7), T cells (n=7), non-cognate (n=8) and cognate ATR (n=9). * (p<0.05) and ** (p<0.001) indicates statistical significance (One-way ANOVA/Kruskal-Wallis nonparametric test). Data generated from two independent experiments.

The ability of ATR to induce redirected lysis *in vivo* was studied by analyzing Raji tumor growth in SICD/beige mice. Raji cells were injected, s.c., at day 0. On day 11, mice were adoptively transferred with 2C T cells i.v. ATR were injected intratumoral on days 11, 14 and 18 (see schematic, Figure 4A). Mice treated with cognate ATR showed reduced tumor growth with statistically significant differences starting at day 14 (Figure 4B). At the termination of the protocol, day 28, mice treated with cognate ATR had the smallest tumor burden compared to mice receiving 2C T cells only, control animals and mice treated with non-cognate ATR. Furthermore, at day 28 already 56% of all control, 25% of T cells only and 20% of non-cognate ATR animals were already dead, whereas all animals from the cognate ATR group were still alive (Figure 4C). Thus we could demonstrate that cognate ATR redirected 2C T cells *in vivo* to engrafted human CD19^+^ Raji cells resulting in redirectional tumor lysis.

## DISCUSSION

In the current report, we describe a nanoparticle-based approach, ATR, to selectively engage antigen-specific T cell and redirect them to kill tumor cells. ATR were generated by coupling either ^pep−^MHC-Ig dimer, or anti-TCR-specific mAb, together with anti-human CD19 mAb onto nanoparticles. ATR stably bound to effector and target cells and induced specific effector-target cell conjugate formation resulting in redirected lysis of human CD19^+^ tumor cells. Finally, ATR demonstrated significant tumor growth inhibition in vivo and prolonged overall survival.

Redirection of antigen-specific T cells has previously been shown in decorating target cells with specific ^pep−^MHC complexes usually following multistep protocols, including immunogenic molecules [10–14]. ATR, to our knowledge, is the first one-step approach redirecting antigen-specific T cell to tumor cells that combines efficacy with optimal half-life particle-size [15] and low toxicity when used in concentrations not exceeding >100-200 μg/ml [16, 17]. Furthermore, antibodies and antibody like complexes have an extremely short *in vivo* half-life [18–20], whereas 10-200nm particles demonstrate prolonged blood circulation times [21–24]. Additionally, particles can be co-functionalized with molecules such as CD47 to further enhance *in vivo* performance [25–27]. Moreover, antibodies are known to diffuse rapidly into the surrounding tissue impacting the overall efficacy [28], however nanoparticles have been described more stably associated with targeted tissues [29]. These well-known properties of nanoparticles were guiding factors in our choice for a nanoparticle based technology.

ATR represent a novel approach for targeted immunotherapy that has several significant potential advantages. First; their ability to selectively engage antigen-specific and not all T cells. Current technologies; BiTEs [30] and engager T cells [31] redirect T cells through targeting CD3. While this has proven to be effective [1–6, 32–34] it also has significant risks such as cytokine storm and severe complications like anaphylaxis [35]. Selective targeting of highly cytotoxic effector T cells using HLA-Ig dimers mitigates against the risk of bystander redirection of unwanted T cells. Second; ATR represent a flexible and cassettable system that can be readily modified by coupling different binding complexes to the nanoparticle. This process is very time efficient and allows for a high degree of personalization. Third; ATR can even be envisioned to be a multi-targeting approach applying different ATR at once or binding different tumor antigens and/or T cell antigen-specificities at the same time utilizing only one ATR preparation.

Since ATR demonstrated tumor growth inhibition and about 60% in vitro killing of tumor cells, tumor evasion might prove a challenge in later clinical translation. This could be tackled utilizing ATR as a combinatory therapy together with standard of care procedures. Therefore, the ATR technology potentially represents an approach with higher impact for tumors with worse prognosis and treatment options. It remains to be seen how ATR will perform in a more immune competent environment and actively recruit T cells to solid and disseminated tumors.

In summary, we believe ATR can be used to develop a novel innovative immunotherapeutic approach for cancers. While the ATR approach provides a very high degree of personalization even imaginable as a “lego like”, off the shelf technology easily adjusted to every patients needs it might also further increase our understanding of tumor immunotherapy through T cell redirection.

## MATERIALS AND METHODS

### Mice and peptides

2C TCR Rag^−/−^ transgenic mice were maintained as heterozygotes by breeding on a C57/BL6 background. PMEL TCR/Thy1^a^ Rag−/− transgenic mice were a gift from Nicholas Restive (National Institutes of Health (NIH), Bethesda, MD). SCID/beige mice (C.B-*Igh*-1b/GbmsTac-*Prkdc^scid^*-Lyst*^bg^*N7) were purchased from Taconic. All mice were maintained according to Johns Hopkins University's Institutional Review Board. Murine peptides SIY (SIYRYYGL) and OVA (SIINFEKL) and human peptide FluM1 (GILGFVFTL) were purchased from Genscript (Piscataway, NJ).

### Preparation of antigen-specific T cells

Murine CD8^+^ T cells were isolated from splenocytes using a mouse CD8^+^ isolation Kit (Miltenyi Biotec). 1×10^6^ CD8^+^ cells were plated on a 96 well round-bottom plate and co-cultured for 7 days at a 1:1 ratio with cognate loaded aAPC in complete RPMI media supplemented with T cell growth factor [36]. On day 7 cells were harvested and particles removed. Viability of >95% was guaranteed by forgone density gradient centrifugation. Human FluM1-specific CD8^+^ T cells were generated as previously published utilizing FluM1 loaded aAPC [37]. The human hybridoma T2 and the Raji B cell lymphoma cell line were obtained from ATCC and cultured in complete RPMI media. Two days before usage cells were split 1:10 to achieve maximal viability.

### Preparation of MHC-Ig dimer

Soluble MHC-Ig dimers, K^b^-Ig and HLA-A2-Ig, were loaded with peptide as described previously [38]. Briefly dimer molecules were loaded with peptide by stripping at alkaline (pH 11.5) or mildly acidic (pH 6.5) conditions, and then refolded in the presence of 40-fold excess peptide and twofold molar excess of human β_2_-microglobulin.[39] Unless otherwise indicated, ^SIY^K^b^, ^OVA^K^b^ and ^FluM1^HLA-A2 (^pep−^MHC) refer to nanoparticle bound MHC-Ig dimer loaded with the indicated peptide.

### ATR and control bead preparation

100 μl of anti-mouse IgG1 Microbeads (Miltenyi Biotec) were co-incubated with 5 μg 1B2 or anti-Vβ17 mAb (Beckman Coulter) or 5 μg peptide loaded ^pep−^MHC-Ig (^SIY^K^b^, ^OVA^K^b^ and ^FluM1^HLA-A2) and 5 μg of an anti-human CD19 mAb (clone HIB19, BD), resulting in an actual 1:1.14 ratio of T cell to tumor binding moieties (data not shown). All control particles were made with 5 μg of one binding complex and 5 μg an appropriate isotype control. To allow binding, particles were incubated at 4°C for >1 hour, washed 3 times (PBS) and eluted in 1 ml PBS; resulting in a 1/10 dilution of the original stock [40,41]. Binding of ^pep−^MHC-Ig and antibodies to particles was routinely analyzed by flow cytometry.

### Flow cytometry

Specificity of human FluM1 specific CTL was determined by FluM1-tetramer (Beckman Coulter) (30 minutes, RT) and anti-CD8 (clone UTHC-4, Sigma) (15 minutes, 4°C) staining. FACS analysis was carried out on a FACSCalibur (BD Biosciences) and analyzed using FlowJo 9.3 software (Treestar).

### Conjugation assays

T2 cells and activated 2C T cells were PKH67 and PKH26 stained as previously published [9]. 1×10^5^ T2 cells were co-cultured at a 1:1 ratio with 2C T cells on a 96 well U-bottom plate together with 50 μl of ATR or control particles (18-24 hours, 4°C). Amount of conjugate formation was determined by flow cytometry, gating on PKH67 and PKH26 double positive cells.

### In vitro redirection killing assay

Cytotoxic activity of redirected T cells was measured by 18-20 hour ^51^Cr release assay using triplicate cultures in V-bottom plates. 2×10^5^/plate tumor cells were pulsed with 200 μCi ^51^Cr (1 hour, 37°C). E:T ratios were 1:1, 2:1, 5:1 and 10:1 on 2000 target cells/well. Each well received 50 μl of ATR or control particles. Percentage of specific lysis was calculated as [(cpm sample - cpm spontaneous release) x100 / (cpm maximum release – cpm spontaneous release)]. For spontaneous release, tumor cells were plated without T cells in complete RPMI media. For maximum release, tumor cells were plated with 0.15% Triton-X-100 (Sigma).

### In vivo tumor inhibition model

On day 0, SCID/beige mice were injected (s.c.) with 5×10^6^ Raji cells into the right flank. On day 11, all mice were adoptively transferred with 5×10^6^ activated 2C T cells and received an intra-tumoral (i.t.) 20μl injection of either cognate (^SIY^K^b^/CD19) or non-cognate (^OVA^K^b^/CD19) ATR (equivalent to 10^10^ ATR) in a total volume of 100 μl of PBS [11]. Treatment was repeated on days 14 and 18. Control groups received only tumor or tumor and 2C T cells. Tumor growth was monitored every other day utilizing a digital caliper. Data was calculated for each individual animal as fold tumor volume (mm^3^) = tumor volume/tumor volume on day 10.

### Statistical analysis

All statistical analyses were performed utilizing GraphPad Prism Software (GraphPad Software Inc., San Diego, CA, USA). * (p<0.05) and ** (p<0.001) indicates statistical significance.

## SUPPLEMENTARY MATERIALS METHODS AND FIGURES



## References

[1] Kellner C, Bruenke J, Stieglmaier J, Schwemmlein M, Schwenkert M, Singer H, Mentz K, Peipp M, Lang P, Oduncu F, Stockmeyer B, Fey GH (2008). A novel CD19-directed recombinant bispecific antibody derivative with enhanced immune effector functions for human leukemic cells. J. Immunother.

[2] Kügler M, Stein C, Kellner C, Mentz K, Saul D, Schwenkert M, Schubert I, Singer H, Oduncu F, Stockmeyer B, Mackensen A, Fey GH (2010). A recombinant trispecific single-chain Fv derivative directed against CD123 and CD33 mediates effective elimination of acute myeloid leukaemia cells by dual targeting. Br. J. Haematol.

[3] Heiss MM, Murawa P, Koralewski P, Kutarska E, Kolesnik OO, Ivanchenko VV, Dudnichenko AS, Aleknaviciene B, Razbadauskas A, Gore M, Ganea-Motan E, Ciuleanu T, Wimberger P (2010). The trifunctional antibody catumaxomab for the treatment of malignant ascites due to epithelial cancer: Results of a prospective randomized phase II/III trial. Int. J. Cancer.

[4] Topp MS, Kufer P, Gökbuget N, Goebeler M, Klinger M, Neumann S, Horst AH, Raff T, Viardot A, Schmid M, Stelljes M, Schaich M, Degenhard M (2011). Targeted therapy with the T-cell-engaging antibody blinatumomab of chemotherapy-refractory minimal residual disease in B-lineage acute lymphoblastic leukemia patients results in high response rate and prolonged leukemia-free survival. J. Clin. Oncol.

[5] Sebastian M, Kiewe P, Schuette W, Brust D, Peschel C, Schneller F, Rühle KH, kNilius G, Ewert R, Lodziewski S, Passlick B, Sienel W, Wiewrodt R, Jäger M, Lindhofer H, Friccius-Quecke H, Schmittel A (2009). Treatment of malignant pleural effusion with the trifunctional antibody catumaxomab (Removab) (anti-EpCAM x Anti-CD3): results of a phase 1/2 study. J. Immunother.

[6] Roskopf CC, Schiller CB, Braciak TA, Kobold S, Schubert IA, Fey GH, Hopfner KP, Oduncu FS (2014). T cell-recruiting triplebody 19-3-19 mediates serial lysis of malignant B-lymphoid cells by a single T cell. Oncotarget.

[7] Chan AC, Carter PJ (2010). Therapeutic antibodies for autoimmunity and inflammation. Nat. Rev. Immunol.

[8] Katz B, Herishanu Y (2014). Therapeutic targeting of CD19 in hematological malignancies : past, present, future and beyond. Leuk. Lymphoma.

[9] Schütz C, Fischer K, Völkl S, Hoves S, Halbritter D, Mackensen A, Fleck M (2009). A new flow cytometric assay for the simultaneous analysis of antigen-specific elimination of T cells in heterogeneous T cell populations. J. Immunol. Methods.

[10] Mous R, Savage P, Remmerswaal EBM, van Lier RAW, Eldering E, van Oers MHJ (2006). Redirection of CMV-specific CTL towards B-CLL via CD20-targeted HLA/CMV complexes. Leukemia.

[11] Lev A, Noy R, Oved K, Novak H, Segal D, Walden P, Zehn D, Reiter Y (2004). Tumor-specific Ab-mediated targeting of MHC-peptide complexes induces regression of human tumor xenografts in vivo. Proc. Natl. Acad. Sci. U. S. A.

[12] King BC, Hamblin AD, Savage PM, Douglas LR, Hansen TH, French RR, Johnson PWM, Glennie MJ (2013). Antibody-peptide-MHC fusion conjugates target non-cognate T cells to kill tumour cells. Cancer Immunol. Immunother.

[13] Savage P, Cowburn P, Clayton A, Man S, McMichael A, Lemoine N, Epenetos A, Ogg G (2002). Induction of viral and tumour specific CTL responses using antibody targeted HLA class I peptide complexes. Br. J. Cancer.

[14] Savage P, Cowburn P, Clayton A, Man S, Lawson T, Ogg G, Lemoine N, McMichael A, Epenetos A (2002). Anti-viral cytotoxic T cells inhibit the growth of cancer cells with antibody targeted HLA class I/peptide complexes in SCID mice. Int. J. Cancer.

[15] Stolnik S, Illum L, Davis SS (1995). Long circulating microparticulate drug carriers. Adv. Drug Deliv. Rev.

[16] Mahmoudi M, Hofmann H, Rothen-Rutishauser B, Petri-Fink A (2012). Assessing the in vitro and in vivo toxicity of superparamagnetic iron oxide nanoparticles. Chem. Rev.

[17] Naqvi S, Samim M, Abdin M, Ahmed FJ, Maitra A, Prashant C, Dinda AK (2010). Concentration-dependent toxicity of iron oxide nanoparticles mediated by increased oxidative stress. Int. J. Nanomedicine.

[18] Portell CA, Wenzell CM, Advani AS (2013). Clinical and pharmacologic aspects of blinatumomab in the treatment of B-cell acute lymphoblastic leukemia. Clin. Pharmacol.

[19] Fournier P, Schirrmacher V (2013). Bispecific antibodies and trispecific immunocytokines for targeting the immune system against cancer: preparing for the future. BioDrugs.

[20] Frankel SR, Baeuerle PA (2013). Targeting T cells to tumor cells using bispecific antibodies. Curr. Opin. Chem. Biol.

[21] Gupta AK, Gupta M (2005). Synthesis and surface engineering of iron oxide nanoparticles for biomedical applications. Biomaterials.

[22] Sawdon A, Peng CA (2013). Engineering antiphagocytic biomimetic drug carriers. Ther. Deliv.

[23] Liu Y., Tan J., Thomas A., Ou-Yang D., Muzykantov V. R. (2012). The shape of things to come: importance of design in nanotechnology for drug delivery. Ther. Deliv.

[24] Sun T, Zhang YS, Pang B, Hyun DC, Yang M, Xia Y (2014). Engineered nanoparticles for drug delivery in cancer therapy. Angew. Chem. Int. Ed. Engl.

[25] Rodriguez PL, Harada T, Christian DA, Pantano DA, Tsai RK, Discher DE (2013). Minimal “Self” peptides that inhibit phagocytic clearance and enhance delivery of nanoparticles. Science.

[26] Bruns H, Bessell C, Varela JC, Haupt C, Fang J, Pasemann S, Mackensen A, Oelke M, Schneck JP, Schütz C (2015). CD47 Enhances In Vivo Functionality of Artificial Antigen-Presenting Cells. Clin. Cancer Res.

[27] Schütz C (2015). CD47 on artificial structures. Aging (Albany NY).

[28] Kwong B, Gai SA, Elkhader J, Wittrup KD, Irvine DJ (2013). Localized immunotherapy via liposome-anchored Anti-CD137 + IL-2 prevents lethal toxicity and elicits local and systemic antitumor immunity. Cancer Res.

[29] Laurent S, Saei AA, Behzadi S, Panahifar A, Mahmoudi M (2014). Superparamagnetic iron oxide nanoparticles for delivery of therapeutic agents: opportunities and challenges. Expert Opin. Drug Deliv.

[30] Baeuerle PA, Reinhardt C (2009). Bispecific T-cell engaging antibodies for cancer therapy. Cancer Res.

[31] Iwahori K, Kakarla S, Velasquez MP, Yu F, Yi Z, Gerken C, Song XT, Gottschalk S (2015). Engager T cells: A new class of antigen-specific T cells that redirect bystander T cells. Mol. Ther.

[32] Aigner M, Feulner J, Schaffer S, Kischel R, Kufer P, Schneider K, Henn A, Rattel B, Friedrich M, Baeuerle PA, Mackensen A, Krause SW (2013). T lymphocytes can be effectively recruited for ex vivo and in vivo lysis of AML blasts by a novel CD33/CD3-bispecific BiTE antibody construct. Leukemia.

[33] Choi BD, Kuan CT, Cai M, Archer GE, Mitchell DA, Gedeon PC, Sanchez-Perez L, Pastan I, Bigner DD, Sampson JH (2013). Systemic administration of a bispecific antibody targeting EGFRvIII successfully treats intracerebral glioma. Proc. Natl. Acad. Sci. U. S. A.

[34] Osada T, Hsu D, Hammond S, Hobeika A, Devi G, Clay TM, Lyerly HK, Morse MA (2010). Metastatic colorectal cancer cells from patients previously treated with chemotherapy are sensitive to T-cell killing mediated by CEA/CD3-bispecific T-cell-engaging BiTE antibody. Br. J. Cancer.

[35] Maus M. V, Haas A. R., Beatty G. L., Albelda S. M., Levine B. L., Liu X., Zhao Y., Kalos M., June C. H. (2013). T cells expressing chimeric antigen receptors can cause anaphylaxis in humans. Cancer Immunol. Res.

[36] Durai M., Krueger C., Ye Z., Cheng L., Mackensen A., Oelke M., Schneck J. P. (2009). In vivo functional efficacy of tumor-specific T cells expanded using HLA-Ig based artificial antigen presenting cells (aAPC). Cancer Immunol. Immunother.

[37] Oelke M., Maus M. V, Didiano D., June C. H., Mackensen A., Schneck J. P. (2003). Ex vivo induction and expansion of antigen-specific cytotoxic T cells by HLA-Ig-coated artificial antigen-presenting cells. Nat. Med..

[38] Dal Porto J, Johansen TE, Catipović B, Parfiit DJ, Tuveson D, Gether U, Kozlowski S, Fearon DT, Schneck JP (1993). A soluble divalent class I major histocompatibility complex molecule inhibits alloreactive T cells at nanomolar concentrations. Proc. Natl. Acad. Sci. U. S. A.

[39] Lebowitz MS, O'Herrin SM, Hamad AR, Fahmy T, Marguet D, Barnes NC, Pardoll D, Bieler JG, Schneck JP (1999). Soluble, high-affinity dimers of T-cell receptors and class II major histocompatibility complexes: biochemical probes for analysis and modulation of immune responses. Cell. Immunol.

[40] Perica K, De León Medero A, Durai M, Chiu YL, Bieler JG, Sibener L, Niemöller M, Assenmacher M, Richter A, Edidin M, Oelke M, Schneck J (2013). Nanoscale artificial antigen presenting Cells for T cell immunotherapy. Nanomedicine.

[41] Perica K, Tu A, Richter A, Bieler JG, Edidin M, Schneck JP (2014). Magnetic field-induced T cell receptor clustering by nanoparticles enhances T cell activation and stimulates antitumor activity. ACS Nano.

