# Correction: Antigen-specific T cell Redirectors: a nanoparticle based approach for redirecting T cells

**DOI:** 10.18632/oncotarget.26018

**Published:** 2018-08-17

**Authors:** Christian Schütz, Juan Carlos Varela, Karlo Perica, Carl Haupt, Mathias Oelke, Jonathan P. Schneck

**Affiliations:** ^1^ Institute of Cell Engineering and Department of Pathology, Johns Hopkins School of Medicine, Baltimore, Maryland, USA; ^2^ Division of Hematology, Department of Medicine, Sidney Kimmel Comprehensive Cancer Center, The Johns Hopkins Hospital, Baltimore, Maryland, USA; ^3^ NexImmune Inc., Gaithersburg, Maryland, USA; ^4^ Current address: Division of Immunology, Paul-Ehrlich-Institut, Langen, Germany

**This article has been corrected:** The correct funding information is given below:

**FUNDING**

This work was in part supported by German Cancer Foundation (Grant no. 70112372), German Research Foundation (SCHU 2681/1-1), HERA Women's Cancer Foundation OSB1 Grant (C.S.) and the National Institutes of Health (P01-AI072677, R01- CA108835 and R21-CA185819), TEDCO/Maryland Innovation Initiative and Neximmune, Inc. MD Biotech Center (J.P.S.).

Original article: Oncotarget. 2016; 7:68503-68512. https://doi.org/10.18632/oncotarget.11785

