# Supplementary material for: Antigen-specific T cell Redirectors: a nanoparticle based approach for redirecting T cells
Source: Oncotarget. 2016 Sep 1;7(42):68503–12. doi: 10.18632/oncotarget.11785 (PMC5356569; doi:10.18632/oncotarget.11785)
Supplement: Supplementary file 1 [file oncotarget-07-68503-s001.pdf]

Antigen-specific T cell Redirectors: a nanoparticle based approach for redirecting T cells

SUPPLEMENTARY INFORMATION

MATERIALS AND METHODS

ATR quality control

Unless otherwise indicated, 0.2×10^6 cells were stained in 50µl $^{pep-}$MHC ATR at 4°C for 45 minutes or 15 minutes for antibody ATR (1B2 and anti-Vbeta17) and washed with 10 volumes of PBS. To detect specific binding of particles to cells, bead-stained cells were secondarily stained with a 1:200 α–mouse IgG1 mAb–PE (Invitrogen) (15 minutes, 4°C).

CD107a activation assay

2C T cells were placed in 96-well round-bottom plates at a density of 2×10^5 cells/well. Prior to stimulation anti–murine CD107a mAb (4 µg/ml), GolgiStop and GolgiPlug (all BD Pharmingen) were added following the manufacturer's protocol. T cells were stimulated with 50 µl ATR in complete RPMI media supplemented with 10% FBS and T cell growth factor. Samples were harvested (5 hours) and immediately analyzed by flow cytometry.

CFSE proliferation assay

Activated 2C T cells were stained with caboxy-succinimidyl-ester (CFSE) (1 µM) following the manufacturer's protocol (Life Technologies). 2×10^5 CFSE labelled 2C T cells were stained with 50 µl ATR (15 minutes, 4°C), washed and cultured in complete RPMI media supplemented 10% FBS and T cell growth factor. On day 3, samples were harvested, washed and analysed by flow cytometry.

RESULTS

Quality control of nanoparticle-based ATR

To evaluate the effective binding of the ATR to their targets, 2C T cells (Supplementary Figure S1 upper panel) or $CD19^+$ tumor cells (Supplementary Figure S1 lower panel), were incubated with ATR and stained with an anti-mouse IgG1 PE mAb. The anti-mouse IgG1 PE mAb antibody is specific for the Fc portion of all molecules on the ATR and thus could be used to visualize ATR bound to cells. Anti-CD19 specific particles (1B2/CD19 and $^{pep-}$MHC-Ig/CD19) bound to T2 cells (Supplementary Figure S1 lower panel). Particles containing TCR specific ligands (1B2, 1B2/CD19, $^{pep-}$MHC-Ig and $^{pep-}$MHC-Ig/CD19 particles) bound to 2C T cells. This data indicates that particles coated with antibody (1B2 mAb) and $^{pep-}$MHC-Ig ($^{SIY}K^b$-Ig) can be generated and are capable of simultaneously binding 2C T cells and tumor cells.

Specificity, stability and ratio dependence of ATR to target cell binding

To set up an optimal protocol for subsequent killing assays, we initially investigated bead-to-cell binding conditions. First, we verified the specificity of a T cell ATR stain and determined the minimal staining time that resulted in a sufficient coating (Supplementary Figure S2A). 2C T cells were incubated with 50µl of either control or ATR (1B2/CD19). While no binding was detected at any time point when stained with control particles (Supplementary Figure S2A, left panel), good binding was detected on 2C T cells incubated with ATR (Supplementary Figure S2A, middle panel). Thereby 15 minutes displayed an optimal staining interval while MFI intensity increased only minimal at later time points. Furthermore, staining of gp-100 specific, transgenic Pmel T cells (Supplementary Figure S2A, left panel) did not show any binding. Experiments utilizing $^{SIY}K^b$-Ig/CD19 ATR also demonstrated antigen–specific binding to 2C T cells (data not shown).

Subsequently, we investigated the stability of ATR to T cell interaction. ATR (1B2/CD19) labeled 2C T cells (Supplementary Figure S2B, black line) were transferred on 37°C after all ATR in excess were washed away. ATR coated 2C T cells were analyzed for decrease of MFI, similar to bead lose (Supplementary Figure S2B). Thus, the data indicate that ATR staining of 2C T cells was stable for ≤60 minutes; that represents a sufficient time interval to provide T to tumor cell interaction in a later killing assay.

We further examined how different bead to cell ratios interfere with an optimal staining outcome. In a first approach we incubated different amounts of 2C T cells with 1B2- (Supplementary Figure S2C, left panel) and T2 tumor cells with CD19-particles (Supplementary Figure S2C, left panel). Best staining was achieved using 0.2×10^6 cells. However, increasing amounts of cells were not correlated with reduced staining intensity. Otherwise, when we varied ATR (1B2/CD19) amounts (Supplementary Figure S2D) 2C T cells (left panel) and T2 tumor cells (right panel) stained with 50µl ATR displayed the most efficient staining. All other ATR amounts resulted in significantly reduced staining intensity.

Finally we investigated the potential of ATR to activated 2C T cells. Therefore cells were incubated with either control particles, $^{OVA}K^b$/CD19 (non-cognate) or $^{SIY}K^b$/CD19 (cognate) ATR and expression of CD107a has been analyzed by flow cytometry (Supplementary Figure S2E). Only 2C T cells co-cultured with cognate ATR demonstrated an activated CD107a$^+$ phenotype. Subsequently, activation of 2C T cells by cognate ATR resulted in a significant increase of proliferation. 2C T cell co-cultures with non-cognate ATR or un-stimulated cultures (control) demonstrated only background proliferation (Supplementary Figure S2F).

SUPPLEMENTARY FIGURES

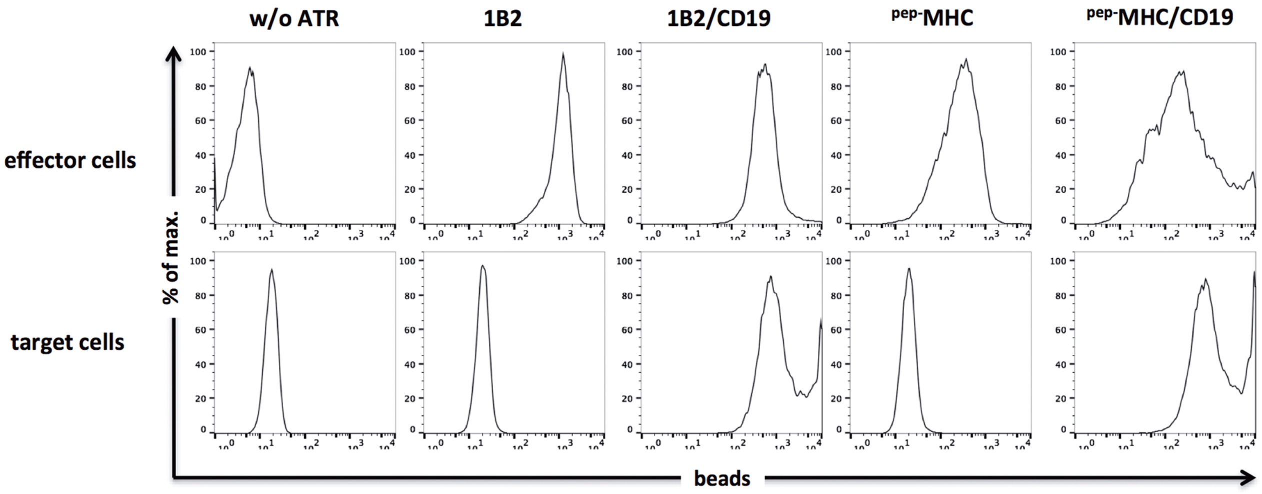

Supplementary Figure S1: Antigen-specific T cell Redirectors (ATR) bind to effector and target cells. Staining control of generated ATR. Effector cells (2C T cells) and target cells (T2 and Raji) were stained with ATR at 4°C for 15 minutes washed and co-stained with anti-mouse-IgG1. $^{pep-}$MHC represents staining of effector or target cells with SIY peptide loaded K^b-Ig dimer generated ATR at 4°C for 45min.

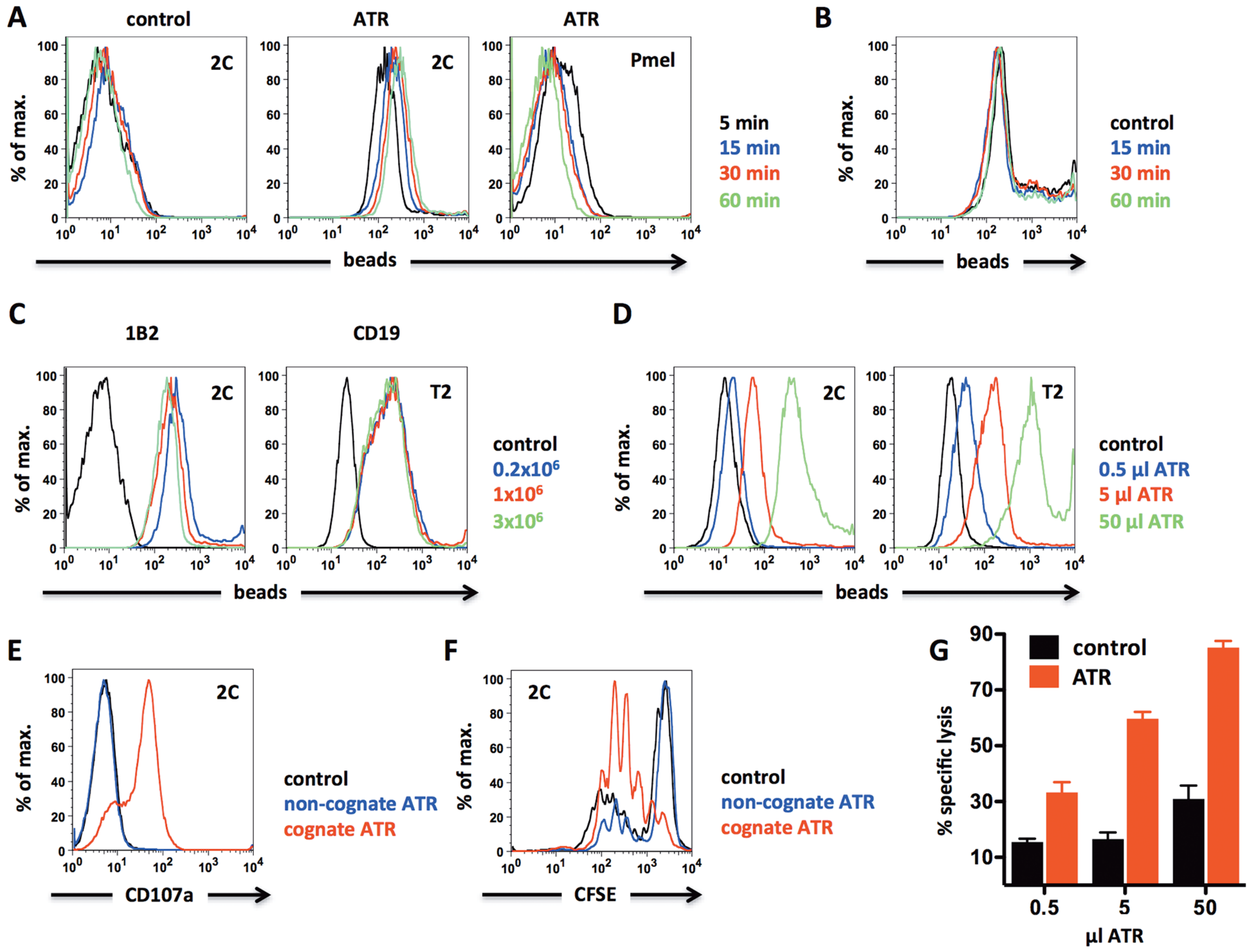

Supplementary Figure S2: Specificity, stability, activation and killing efficacy of ATR binding to target and effector cells. A. To test the specificity of ATR (1B2/CD19) binding 2×10^5 2C and Pmel T cells were incubated with 50 µl of ATR at 4°C for the duration indicated. **B.** To test the stability of ATR binding 2×10^5 2C T cell were incubate at 4°C for 15 minutes with 50 µl ATR (control), washed and subsequently transferred at 37°C. After indicated time points cells were analyzed for loose of ATR. ATR binding of 2C T cell and $CD19^+$ tumor cells (T2) was investigated relating the impact of bead:cell ratio on staining intensity. Therefore, either different amounts of cells, as indicated **C.** were stained with 50 µl ATR or **D.** 2×10^5 2C T cells were stained with different amounts of ATR, as indicated. For detection of ATR cells were washed and secondary stained with anti-mouse IgG1. **E.** To determine the activation potential of ATR activated 2C T cells were incubated with 50 µl of either control particles, OVA peptide loaded K^b/CD19 (non-cognate) or SIY peptide loaded K^b/CD19 (cognate) ATR. After 4 hours expression of CD107a on cells was analyzed. **F.** Same ATR (50 µl) were bound to CFSE stained 2C T cells, washed and co-cultured for 3 days. Proliferation was determined by CFSE dilution compared to non-stimulated cells (control). **G.** To investigate ATR killing efficacy, specific lysis of $CD19^+$ Raji cells was analyzed in a ^{51}Cr-release killing assay utilizing a 10:1 E:T ratio. 2×10^5 2C T cells were incubated with different amounts of ATR; 5×10^7/ml (0.5µl), 5×10^8/ml (5µl) and 5×10^9/ml (50µl).
